# Complementary and alternative medicine use: Results from a descriptive study of pregnant women in Udi local Government area of Enugu state, Nigeria

**DOI:** 10.1186/s12906-017-1689-0

**Published:** 2017-04-04

**Authors:** Jane-lovena Onyiapat, Chinyelu Okafor, Ijeoma Okoronkwo, Agnes Anarado, Ekene Chukwukelu, Ada Nwaneri, Pat Okpala

**Affiliations:** 1 Department of Nursing Sciences, University of Nigeria, Enugu Campus, Enugu, Nigeria; 2 Department of Health Administration & Management, University of Nigeria, Enugu Campus, Enugu, Nigeria; 3 Department of Chemical Pathology, College of Medicine, University of Nigeria, Enugu Campus, Enugu, Nigeria

**Keywords:** Complementary and alternative medicine, Use, Pregnant women

## Abstract

**Background:**

The use of CAM by pregnant women is very popular in developed countries. The trend is increasing globally and lack of evidence of safety particularly when used during pregnancy may lead to complications. Pregnancy is a vulnerable period especially during the first trimester. There is scarcity of empirical evidence on CAM use particularly among women in Udi LGA of Enugu State and South East Nigeria. Moreover, studies carried out in Nigeria have been limited to herbal medicine use, which is one aspect of CAM. This study was designed to obtain information on the use of Complementary and Alternative Medicine among pregnant women.

**Methods:**

The study was a cross sectional descriptive survey of 396 pregnant women systematically drawn from twenty political wards in Udi Local Government Area (LGA) of Enugu State. Interviewer administered questionnaire developed by the researchers was used for data collection. Data were analyzed using descriptive statistics.

**Results:**

Majority (82.1%) of the pregnant women in Udi LGA used CAM during pregnancy out of which 53.8% had used CAM in previous pregnancies. CAM used ranges from one single type to sixteen different types with biological products eg, herbal tea, herbal mixture being the most commonly used CAM. Whereas most (89.5%) of the CAM used by pregnant women was consumed orally, approximately half of the pregnant women used CAM together with conventional medicine.

**Conclusion:**

The use of CAM by women during pregnancy was high in Udi LGA. Therefore, maternity care providers especially midwives need to elicit CAM commonly used by women during pregnancy and counsel them appropriately for best care and safety. Researchers should focus on establishing the efficacy of CAM products.

**Electronic supplementary material:**

The online version of this article (doi:10.1186/s12906-017-1689-0) contains supplementary material, which is available to authorized users.

## Background

Complementary and alternative medicine (CAM) use in Nigeria is becoming more popular [1–3], as in many other countries of the world. Globally, the prevalence of CAM use range from 30 to 75% [4]. CAM includes various approaches/techniques outside the western conventional medicine that are used to prevent and/or treat illnesses/diseases as well as promote health. CAM has been defined by the National Center for Complementary and Alternative Medicine [NCCAM] [5] as a group of medical and health care systems, practices and products that are not currently part of conventional medicine. When unconventional approach and/or product are used together with conventional medicine, it is said to be complementary; but when it is used in place of conventional medicine, it becomes an alternative medicine. Therefore CAM is an umbrella term used for both complementary and alternative health care practices.

The role played by CAM during pregnancy, can no longer be dismissed as a faddish whim accessed by poorer people. The trend is increasing globally and lack of robust evidence of safety, particularly when used in a vulnerable population such as pregnant women is not a deterrent to CAM use [6]. Pregnancy is a vulnerable period especially the first trimester. This is the period of organogenesis when rapid cell division and migration takes place. Any alteration during the process of mitosis and meiosis could result to serious pregnancy complications that may affect both mother and baby adversely [6]. The pregnancy complications may consequently result to maternal and perinatal morbidity (which may be temporal or permanent), and mortality. Pregnancy being a vulnerable period may be compounded in a rural community like Udi LGA where there is lack of functional comprehensive conventional medical services.

Recently, just before this study was carried out, the researchers conducted a focus group discussion (FGD) on CAM use among pregnant women in Ezeagu LGA. Ezeagu LGA is a neighbouring LGA to Udi LGA, formerly in Udi LGA. The focus group discussion involved six pregnant women who were engaged in discussions of what women use during pregnancy, how and why pregnant women use such practices among others. The focus group discussion revealed that pregnant women use a plethora of products, services and practices under the umbrella of three main categories of CAM including biological products, alternative medical systems and mind body interventions (spiritual therapy) from the NCCAM [5] classification of CAM. This present study will concentrate on these three main categories of CAM.

Prior to pregnancy, women may have used CAM and may continue to use or initiate using CAM during pregnancy and for parturition, regardless of the lack of scientific evidence concerning its safety [6] and efficacy. The use of CAM by pregnant mothers is very popular in developed countries and relatively common in developing countries [7, 8]. Globally, the use of CAM among pregnant women ranges from 7 to 55% [9] and up to 96% [7].

A significant use of CAM has been recorded among: English pregnant women, (57.8%) including herbs and spiritual therapy [10]; Australian Women, (36 to 73%), and the most prevalent being herbs such as castor oil and ginger [7, 11]; Iranian women, (30.8%), including ammi, saatar and sweet basil [8]; South African women (100%) ranges from traditional medicine, faith healing and “Dutch medicine” [12] and Zambian women, (21 to 30%) [13]. In Nigeria, Fekeye et al., [14] and Tamuno et al., [15] recorded 67.5% and 31.4% of herbal medicine use respectively among pregnant women, with ginger and garlic being the most popular herbs [15]. More than half of the pregnant women who used CAM use multiple therapies [11]. Although most herbs used were in no particular pattern in relation to gestational age, most of the pregnant women who used herbs did so during first trimester [7, 15]. CAM use may also be common after the first trimester [12]. About 75% of pregnant women do not disclose use to their conventional maternity care providers and most self medicate [10]. Some of them use CAM concomitantly with conventional medicine [8, 12, 15].

The present study was conceived based on the scarcity of empirical literature on CAM use particularly among women in Udi LGA of Enugu State and South East Nigerian women in general during pregnancy. Unfortunately, Studies carried out in Nigeria were limited to herbal medicine [14, 15], (an example of a class of CAM), use during pregnancy and were mainly hospital based. This present study is community-based and revealed the prevalence, types of CAM used by women during pregnancy under the three selected categories of CAM and patterns of CAM use. This study provided some fundamental information on the most common CAM practices during pregnancy in Udi LGA.

## Methods

### Design and area of study

Cross sectional descriptive survey design was used for the study. The study was carried out in Udi Local Government Area (LGA) of Enugu State, Nigeria. Udi is one of the LGA located in Enugu West senatorial district with its head quarter at Udi town. Politically, Udi LGA is sub-divided into North and South with development centers in both subdivisions. Overall, Udi LGA comprises twenty political wards formed by the different towns in Udi LGA. As at 2006, Udi LGA had a population of 238,305 comprising 117,914 males and 120,391 females (National Population Commission [NPC] [16]. The people of Udi LGA are mainly subsistent farmers, civil servants and palm wine tappers.

### Population of study

The population of study comprised pregnant women between the ages of 15 to 49, who registered for antenatal care in the various health centers in Udi LGA from July 2012 to December, 2012. The total number of women who fell within this category was estimated at 1204 [17].

### Sample and sampling procedure

A sample size of 384 pregnant women was determined using the formula for calculating sample size [18] as follows: n = z^2^P(1-P)⁄d^2^. Applying the adjustment formula, q = n/1-f the sample size (*n* = 384) was further increased to 396 in order to make room for non response rate.

Systematic sampling method was used to proportionately select 396 pregnant women from the list of pregnant women generated from the twenty political wards in Udi LGA. One out of every three pregnant women was selected for the study. The first respondent was selected at random. Other selections were made at intervals of three until a sample of 396 pregnant women was obtained.

### Ethical consideration

Ethical clearance for the study was obtained from the Enugu State Ministry of Health Ethical Committee. Written administrative consent was obtained from the chairman, Udi LGA. Oral informed consent was obtained from the political ward heads while written informed consent was also obtained from the respondents before administration of the questionnaire. Confidentiality of respondents’ information was strictly observed.

### Data collection

Questionnaire developed by the researchers was the tool used for data collection. The 16 item pretested questionnaires comprised mainly closed ended questions and very few open ended questions (Additional file 1). The tool was in two sections; section A sought information on demographic profile of the respondents while section B obtained information on CAM use, types and patterns of CAM use. The questionnaire was drawn based on extensive literature search on CAM, and information on CAM use obtained from focus group discussion. The questionnaire was interviewer administered to accommodate literate and illiterate respondents. The questions in the questionnaire were interpreted in the local (Igbo) language to the selected respondents and their responses were recorded into the questionnaire.

### Data analysis

Data collated were analyzed using descriptive statistics including frequencies and percentages. This was done using the computer programme graph pad prism version 5.03. The results were presented on Tables.

## Results

The data generated including the demographic characteristics of the respondents were presented using tables. The entire 396 questionnaire was appropriately filled and returned.

### Socio demographic profile of respondents

Out of 396 respondents, 221 (55.8%) were within the ages of 25 and 34, 54 (13.6%) fell within the ages of 35 and 44. The mean age of the respondents was 27.8 (SD ± 6.4).

The respondents were mostly married 379 (95.7%) and majority 307 (77.5%) attained secondary education. The major occupation of the respondents was petty trading, 181 (45.7%). With regards to parity, 328 (82.8%) had been pregnant one to four times with 58.8% in the third trimester of the current pregnancy. General condition of the respondents was reported to be good 221 (55.8%). (see Table 1).

**Table 1:** Cross tabulation of demographic profile of users and non users of CAM (*n* = 396)

Parameter	User (%)	Non user (%)	Total (%)
*Age*			
15–24 yrs	84 (21.2)	37 (9.3)	121 (30.5)
25–34 yrs	193 (48.7)	28 (7.1)	221 (55.8)
35–44 yrs.	48 (12.1)	6 (1.5)	54 (13.6)
Total	325 (82.1)	71 (17.9)	396 (100.0)
*Marital status*			
Married	*313 (79.0)*	66 (16.7)	371 (95.7)
Not married	12 (3.0)	5 (1.3)	17 (4.3)
Total	325 (82.1)	71 (17.9)	396 (100.0)
*Educational level*			
Primary	36 (9.1)	5 (1.3)	41 (10.4)
Secondary	252 (63.6)	55 (13.9)	307 (77.5)
Tertiary	37 (9.3)	11 (2.8)	48 (12.1)
Total	325 (82.1)	71 (17.9)	396 (100.0)
*Occupation*			
House wife	28 (7.1)	13 (3.3)	41 (10.4)
Petty trader	152 (38.4)	29 (7.3)	181 (45.7)
Artisan/ learnt trade	116 (29.3)	21 (5.3)	137 (34.6)
Professional	29 (7.3)	8 (2.0)	37 (9.3)
Total	325 (82.1)	71 (17.9)	396 (100.0)
*Parity*			
1–4	262 (66.2)	66 (16.6)	328 (82.8)
5–8	63 (15.9)	5 (1.3)	68 (17.2)
*Trimester*			
First	14 (3.5)	10 (2.5)	24 (6.0)
Second	115 (29.1)	24 (6.1)	139 (35.2)
Third	196 (49.5)	37 (9.3)	233 (58.8)
*Health condition*			
Satisfactory	150 (37.9)	25 (6.3)	175 (44.2)
Good	175 (44.2)	46 (11.6)	221 (55.8)
Total	325 (82.1)	71 (17.9)	396 (100.0)

### Prevalence of CAM use

Table 2 reveals that out 396 respondents, 325 (82.1%) were using CAM during pregnancy, 175 (53.8%) had used CAM in previous pregnancies while 150 (46.2%) were first time users.

**Table 2:** Prevalence of CAM use among pregnant women in Udi LGA (*n* = 396)

CAM use	Frequency (%)
Non user	71 (17.9%)
User	325 (82.1%)
Total	396 (100%)
^a^User during previous pregnancies (*n* = 325)	175 (53.8%)
^a^First time users (*n* = 325)	150 (46.2%)
Total	325 (100%)

^a^users of CAM at the time of the study

The result in Table 3 indicates that 225 (69.2%) used one to four different types of CAM while 77 (23.7%) of the respondents used five to eight different types of CAM and only 3 (0.9%) use thirteen to sixteen various types of CAM. The mean number of CAM used was 4.1 (SD ± 2.58).

**Table 3:** Number of CAM used by pregnant women in Udi LGA. (*n* = 325)

Number of CAM used by pregnant women	Frequency (%)
1–4	225 (69.2%)
5–8	77 (23.7%)
9–12	20 (6.2%)
13–16	3 (0.9%)
Total	325 (100)

### Categories of CAM used by pregnant women

Table 4 indicates that biological products 250 (76.9%) and alternative medicine 232 (71.4%) were the class of CAM highly favoured by the respondents. Spiritual therapy 98 (30.2%) was the least prevalent among the respondents. Herbal mixtures 110 (33.8%), herbal tea *“mvuruinu”* 102 (31.4%) and olive oil 79 (24.3%) were biological products predominantly used by the respondents. Traditional Birth Attendant 197 (60.6%), traditional external cephalic version 82 (25.2%) and folk remedies 66 (20.3%) were common alternative medicine used by the respondents while prayer/faith healing 78 (24%), vision 25 (7.7%) and pregnancy ritual 21 (6.5%) were highly rated under the class of spiritual therapy.

**Table 4:** Categories and specifics under the categories of CAM used by pregnant women

Categories and specifics of CAM	Frequency (%)^a^
Biological products^b^	250 (76.9)
^c^Herbal mixture	110 (33.8)
^c^Herbal tea *“mvuruinu”*	102 (31.4)
^c^Olive oil	79 (24.3)
Alternative medicine^b^	232 (71.4)
^c^Traditional Birth Attendant patronage	197 (60.6)
^c^Traditional external cephalic version	82 (25.2)
^c^Folk remedies	66 (20.3)
Spiritual therapy^b^	98 (30.2)
^c^Prayer/faith healing	78 (24.0)
^c^Vision	25 (7.7)
^c^Pregnancy ritual	21 (6.5)

^a^Respondents ticked more than one option; ^b^categories; ^c^specifics

### Pattern of use of CAM among pregnant women

The result in Table 5 reveals that the most frequent pattern (route) of administration of CAM is orally 291 (89.5%), followed by consultations/adherence to the advice of Traditional Birth Attendants 164 (50.5%) and topical application 101 (31.1%). Others include body manipulation 98 (30.2%), spiritual cleansing and deliverance 89 (27.3%) and insertions into the vagina 29 (8.9%).

**Table 5:** Pattern of administration or consumption of CAM. *n* = 325

Pattern of administration	Frequency (%)^a^
Oral administration	291 (89.5)
Consultations/adherence to Traditional Birth Attendance’s advice,	164 (50.5)
Body manipulations	98 (30.2)
Topical application	101 (31.1%)
Spiritual consultations, cleansing and deliverance	89 (27.3)
Insertion into the vagina	29 (8.9)

^a^Respondents ticked more than one option

Table 6 shows that the respondents used their CAM mainly during the third trimester 89 (27.4%) and throughout pregnancy 81 (24.9%). Women used CAM least during the first & second 8 (2.5%) and second & third 32 (9.8%) trimesters.

**Table 6:** Use of CAM by stage of pregnancy. (*n* = 325)

Stage of pregnancy	Frequency (%)
Third trimester only	89 (27.4)
Throughout pregnancy	81 (24.9)
Second trimester only	66 (20.3)
First trimester only	49 (15.1)
Second and third trimester	32 (9.8)
First and second trimester	8 (2.5)
Total	325 (100)

As shown in Table 7, 166 (51.1%) used only CAM while 159 (48.9%) used CAM and conventional medicine together during pregnancy.

**Table 7:** Use of CAM and conventional medicine. *n* = 325

Use of CAM/conventional medicine	Frequency (%)
use only CAM	166 (51.1)
use combination of CAM and conventional medicine	159 (48.9)
Total	325 (100)

## Discussion

### Prevalence of CAM use among pregnant women in Udi LGA

The findings of this study revealed that majority of the pregnant women in Udi LGA used CAM. It is inferred from the findings that the number of pregnant women in Udi LGA who used CAM during pregnancy is estimated at 1970 per annum. That is 821 users of CAM per 1000 pregnant women per annum. Slightly more than half of the CAM users had a record of previous use while approximately 46% were first time users of CAM. This prevalence falls within the range (30% - 96%) reported in literature among pregnant women [7, 8, 10, 11] and among the general adult population [4]. This finding also confirms the report that 80% of the population in Africa depend on CAM for their primary health care needs [19].

However, this result is higher than previous findings in Nigeria which reported a prevalence of 67.5% [14] and 31.4% [15] on herbal medicine use among pregnant women in Nigeria. This high prevalence may be attributed to the wider coverage of unconventional health care systems in this study. The present study was carried out in a rural community, while the previous studies were health facility based, conducted in Northern part of Nigeria and limited to herbal medicine use only.

The number used by each user of CAM ranged from one single type to sixteen different types with a mean of 4.1. This implies that for every pregnant woman in Udi LGA who used CAM engaged in approximately four different types of CAM. This finding is higher than what was reported in previous study [8] which recorded a mean of 1.4 per each CAM user. The variation may be as a result of what is included as CAM in the study.

### Categories of CAM used by pregnant women in Udi LGA

Biological products and alternative medicine were the most frequent category of CAM used by women during pregnancy. Herbal mixtures and herbal tea (*mvuruinu*) were the most commonly used among the biological products while engaging the services of Traditional Birth Attendants (TBA) and traditional external cephalic version gained popularity among the alternative category. This finding is in line with previous studies which identified herbal remedies as the most commonly used CAM among pregnant women [11, 12, 20]. It also agrees with the findings of WHO [4] where Traditional Birth Attendants were observed to have assisted in majority of births in several African countries including Nigeria.

Spiritual therapy was another significant class of CAM employed by women during pregnancy. Close to one quarter of the pregnant women who used CAM engaged in prayers as a form of CAM therapy. Other religious practices such as vision, cleansing/deliverance and pregnancy ritual were also reported by users of CAM. Faith healing has been reported in previous study as one of the commonest CAM used by pregnant women in South Africa [12]. Spirituality is the most ancient healing practices [21]. The relative high prevalence of prayer/faith healing among pregnant women may be attributed to the African ancestry affinity to spiritual and transcendental phenomenon. Considering spirituality in the light of holistic philosophy of CAM, it involves relationship with oneself, with others and with a higher power.

### Patterns of CAM use among pregnant women in Udi LGA

The major route of consumption of CAM was orally, followed by topical application. Almost 90% of CAM used by pregnant women was consumed orally. Previous research reported similar findings of 99.1% for oral consumption of herbs among pregnant women in South Iran [8].

Half of the pregnant women that used CAM had to either consult and/or visit the CAM practitioners to obtain the CAM therapies. Very few visited deities in order to enjoy the CAM practices required. These findings support reports of past studies which stated that women consulted CAM practitioners during pregnancy for some pregnancy related reasons [11, 13, 22]. The result of this study also identified that a small but significant number of women used their CAM through direct insertion of remedies such herbs in to the vagina.

The most frequent stage of pregnancy during which women used CAM was the third trimester and nearly the same number used their CAM throughout the period of pregnancy. This finding is in line with the reports of some previous studies which identified third trimester as the stage when pregnant women frequently use CAM [7, 23, 24]. However, this does not correspond with the findings of some other studies which recorded that use of herbs among pregnant women occurred mostly during the first trimester [8, 15]. This difference may be related to ethnicity, the women’s knowledge on CAM and its properties and what were perceived to constitute CAM therapies. Different ethnic groups have different belief system and practices peculiar to them. Ethnic differences exist in the use of CAM among pregnant women [7].

Although more than half of the women do not use CAM in combination with conventional medicine, a little lower than half use CAM concomitantly with conventional medicine. This result agrees with the report of previous study [14] where more than half of the respondents did not agree that herbs should be used in combination with conventional medicine. The finding also conforms to similar studies [8, 12] where some pregnant women used CAM in addition to conventional health practices. The implication of using CAM in combination with conventional medicine is that, there may be possible interaction between CAM remedies and conventional medicine and such interaction could be detrimental to the body. Moreover, in Nigeria, there have been reports of some ready to use herbal products being contaminated with heavy metals such as lead [25, 26]. This contamination could lead to liver and kidney overload and consequently failure of these organs.

The major strength of this study lies in the fact that it is the fundamental CAM study among pregnant women that included other classes of CAM and not just limited to herbal medicine. The interviewer administered questionnaire ensured completeness of data with 100% return rate. However, the findings of this study are limited and may not be generalized to all pregnant women in Nigeria. This is because the study setting and sample size do not form a national representative sample of pregnant women in Nigeria.

## Conclusion

This study was designed to determine the use of complementary and alternative medicine among pregnant women in Udi LGA. Findings revealed a high prevalence of CAM use among pregnant women in Udi LGA, more than that reported in similar studies carried out in Northern part of Nigeria. Most pregnant women in Udi LGA used some form of CAM including biological products, of which herb is the most common. Most of the CAM therapies were consumed orally and concomitantly with conventional medicine. Therefore maternity care providers especially midwives need to be conversant with CAM commonly used by women during pregnancy and rid themselves off misconceptions about CAM. There is also need to routinely include pregnant women use of CAM history during antenatal booking and other visits.

## Additional file

Additional file 1: Questionnaire on use of cam by pregnant women. (DOCX 19 kb)

## Abbreviations

CAM: Complementary and Alternative Medicine
LGA: Local Government Area
NCCAM: National Center for Complementary and Alternative Medicine
