# Supplementary material for: Complementary and alternative medicine use: Results from a descriptive study of pregnant women in Udi local Government area of Enugu state, Nigeria
Source: BMC Complement Altern Med. 2017 Apr 4;17:189. doi: 10.1186/s12906-017-1689-0 (PMC5379706; doi:10.1186/s12906-017-1689-0)
Supplement: Additional file 1: — Questionnaire on use of cam by pregnant women. (DOCX 19 kb) [file 12906_2017_1689_MOESM1_ESM.docx]

**QUESTIONNAIRE ON USE OF COMPLEMENTARY AND ALTERNATIVE MEDICINE (CAM) AMONG PREGNANT WOMEN IN UDI LGA, ENUGU STATE**

This questionnaire is designed to find out to what degree pregnant women employed complementary and alternative medicine in their lives. It is only a research work. The answer you give to the questions here will in no way influence how you are treated as an individual. The findings of this research will help us to understand our women better and improve the care that we render to them. The findings will also be disseminated through publication in electronic and print media. I implore you to give as honest an answer to each question as possible. I guarantee your confidentiality.

**SECTION A: Demographic data**

Please circle and fill in as applicable

1. Age as at last birthday ---------------------------------------
2. Marital status
3. Married
4. Not married
5. widow
6. divorced /separated
7. Highest level of education attended:
8. No formal education
9. Primary education
10. Secondary education
11. Tertiary education
12. What do you do for a living (occupation)? ------------------------------------------
13. What is your religion?
14. Christian religion
15. Islamic religion
16. Traditional religion
17. How many times have you been pregnant? --------------------------------
18. How old is this current pregnancy? -----------------------------------------

**SECTION B:**

1. How would you rate your overall health?
2. Satisfactory
3. Good
4. Not good
5. Seriously sick

Objective one: To determine the prevalence of CAM use among pregnant women in Udi LGA.

1. Have you used any medicine or remedy (traditional medicine) other than that given to you in a hospital by a heath care professional since you discovered you were pregnant?
2. Yes
3. No
4. Are you currently using any traditional medicine or remedy other than that given to you in a hospital by a medical practitioner since you discovered that you were pregnant?

a. Yes

b. No

Objective two: To ascertain the various classes of CAM women in Udi LGA use during pregnancy.

1. If yes to 9 and 10 above, please tick all the traditional medicine you have used and/ or using and the route of administration.

|  | Traditional medicine/ products | Oral consumption | Topical application | Insertion |  |
| --- | --- | --- | --- | --- | --- |
|  | Plants products |  |  |  |  |
|  | Ginger |  |  |  |  |
|  | Aloe Vera |  |  |  |  |
|  | Pumkin seed |  |  |  |  |
|  | Morringer seed |  |  |  |  |
|  | Garlic |  |  |  |  |
|  | Unripe whole fruits |  |  |  |  |
|  | Cowpea leaves |  |  |  |  |
|  | Herbal mixtures |  |  |  |  |
|  | Herbal tea |  |  |  |  |
|  | Aboshi |  |  |  |  |
|  | Vegetable (Ariraa) |  |  |  |  |
|  | Olive oil |  |  |  |  |
|  | Castor oil |  |  |  |  |
|  | Nutritional supplement |  |  |  |  |
|  | Bitter cola |  |  |  |  |
|  | Animal products | Drinks it thru the mouth | Robs it on the body | Massage/ manipulation | Consultations with provider |
|  | Wall gecko |  |  |  |  |
|  | Honey |  |  |  |  |
|  | Hitting the pelvic region with a specific animal bone |  |  |  |  |
|  | Alternative medicine |  |  |  |  |
|  | Folk remedies |  |  |  |  |
|  | Natural remedies |  |  |  |  |
|  | Traditional birth attendant |  |  |  |  |
|  | Traditional external cephalic version |  |  |  |  |
|  | Spiritual therapies | Fasting | Recitation | Incantation | Visits |
|  | Faith/ prayer |  |  |  |  |
|  | Vision |  |  |  |  |
|  | Meditation |  |  |  |  |
|  | Divination |  |  |  |  |
|  | Cleansing |  |  |  |  |
|  |  |  |  |  |  |

1. Are there other traditional medicine or remedies/products/ not listed above which you have used and/or using?
2. Yes
3. No
4. If yes, please specify--------------------------------------
5. Do you hope to use such traditional medicine or remedies again during subsequent pregnancy
6. Yes b. No C. may or may not use

Objective three: to identify patterns of CAM use among pregnant women in Udi LGA.

1. At what stage of pregnancy did you use the traditional medicines or remedies you ticked?
2. 0 to 3 months d. 0 to 6 months
3. 4 to 6 months e. 4 to 9 months
4. 7 to 9 months f. 0 to 9 months
5. Which of the following statement best explain how you have been using the traditional medicines or remedies and medicine given to you in the hospital by a medical practitioner?
6. You use only one of the medicine at a time
7. You use both the traditional medicine or remedies and medicine given to you in the hospital together
